# Patient outcomes in Anteromedial osteoarthritis patients over 80 years old undergoing Oxford Unicompartmental knee Arthroplasty in China

**DOI:** 10.1186/s12891-020-03474-0

**Published:** 2020-07-08

**Authors:** Jingbo Cheng, Mingli Feng, Guanglei Cao, Zheng Li, Shuai An, Shibao Lu

**Affiliations:** Department of Orthopaedics, Xuanwu Hospital, Capital Medical University, Changchun Ave 45, Xicheng District, Beijing, 100053 China

**Keywords:** Oxford Unicompartmental knee Arthroplasty, 80 years old, Perioperative complication, Restoration of function, Safety

## Abstract

**Background:**

The use of Oxford Unicompartmental Knee Arthroplasty (UKA) has increased rapidly in both Western and Asian populations, with excellent functional outcomes and high patient satisfaction. While previous evidence regarding clinical outcomes and survival rates after Oxford UKA was based on studies in Western populations, the results may be different in Asian patients. The relevance of age for postoperative function after Oxford UKA also remains unclear. Hence, the aim of our study was to clarify the effectiveness and safety of Oxford UKA in Asian patients aged over 80 years.

**Methods:**

A retrospective review was performed and included 195 patients (209 knees) who underwent an Oxford UKA between June 2015 and July 2018. We divided the patients into three groups by age: Group 1, 60–69 years; Group 2, 70–79 years; and Group 3, over 80 years. We used the Hospital for Special Surgery (HSS) score and Western Ontario and McMaster (WOMAC) Universities Osteoarthritis Index score to evaluate the general condition of the patients’ knees before surgery and at last follow-up. We also recorded perioperative and short-term complications.

**Result:**

Group 1 consisted of 60 patients (60 knees); Group 2, 70 patients (79 knees); and Group 3, 65 patients (70 knees). The mean follow-up was 21.34 ± 12.04, 22.08 ± 11.38, and 21.76 ± 10.20 months in groups 1, 2, and 3, respectively. At last follow-up, the patients in Group 3 showed lower function scores compared to groups 1 and 2 (*P* < 0.05), but the HSS scores and the WOMAC scores were significantly improved in all three groups. In terms of perioperative and other complications, the three age groups did not differ significantly.

**Conclusion:**

Oxford UKA is an effective and safe treatment for osteoarthritis, even in elderly patients in China. Elderly patients have lower knee function scores than younger patients. However, the knee joint pain of the elderly patients was relieved and function improved compared to the preoperative condition.

## Background

Oxford unicompartmental knee arthroplasty (UKA) is one of most effective surgical procedures for the treatment of isolated medial compartment osteoarthritis. Compared to total knee arthroplasty (TKA), UKA (including both fixed-bearing UKA and mobile-bearing UKA) can provide better physiological function, quicker recovery, shorter hospital stay, and fewer perioperative complications, especially for early arthritis [1, 2]. Recently, the use of Oxford UKA has increased rapidly around the world, and the effectiveness and safety of a minimally invasive surgical approach for Oxford UKA has demonstrated good-to-excellent long-term follow-up in Western populations [3, 4]. Today, with the improvement in medical standards, the phenomena surrounding aging is becoming increasingly obvious worldwide. Many elderly patients cannot undergo surgery because of its high risk. Due to the potential reduction of morbidity and mortality compared to TKA, UKA may represent an interesting solution for elderly patients when the disease is limited to medial femorotibial disease.

Asian populations have different lifestyles, such as squatting and sitting on the floor, from those of Western populations. Therefore, Asian populations need more range of motion (as they often flex the knee to more than 120 degrees) to perform daily activities [5, 6]. A previous study also reports a substantially higher bearing dislocation rate in Asian populations [7], which may relate to the lifestyle-related high degrees of knee flexion. While previous evidence regarding clinical outcomes and survival rates after Oxford UKA were based on studies in Western populations, results may be different in Asian patients. Furthermore, previous studies did not compare different age groups. Thus, the relevance of age in postoperative function after Oxford UKA remains unclear. Hence, the aim of our study was to clarify the effectiveness and safety of Oxford UKA in Asian patients aged over 80 years.

## Methods

### Participants

We retrospectively reviewed 195 knee osteoarthritis patients who underwent medial Oxford UKA (Oxford Unicompartmental Phase 3, Biomet, Warsaw, IN, USA) between June 2015 and July 2018. The study protocol was reviewed and approved by the hospital institutional review board. All patients provided informed consent for participation in the study. All patients were diagnosed with anteromedial osteoarthritis (AMOA) of the knee based on history, physical examination, and radiographs. Before surgery, all patients had standard X-ray views taken: anteroposterior (AP) and lateral radiographs; full-length standing, weight-bearing AP, and lateral radiographs; and patella tangential view radiographs. Magnetic resonance imaging (MRI) was also done to evaluate the ligament, meniscus, and lateral compartment.

The indication criteria for UKA were: older than 55 years; a correctable varus deformity (varus less than 15 degrees); flexion deformity less than 15 degrees; intact ligaments, especially the anterior cruciate ligament (ACL) and medial collateral ligament (MCL) which should be functionally normal; an intact lateral compartment; proper range of motion (at least 0–100 degrees); and no inflammatory disease. We observed the stage of deterioration of the ACL intraoperatively. If the ACL deficiency was less than grade 2 we performed an Oxford UKA (ACL grades: 1. Normal; 2. Loss of synovial covering, usually starting distally; 3. Longitudinal splits in the substance of the exposed ligament; 4. Friable and fragmented with stretching and loss of strength of the collagen bundles; 5. Absent or ruptured).

Exclusion criteria: The lateral side of the patellofemoral joint exhibited bone loss with eburnation and longitudinal grooving; absent or severely damaged ACL (or PCL or MCL).

### Surgical technique and perioperative management

The same group of surgeons performed all the operations. The patient was placed in the supine position and a tourniquet was applied to the proximal thigh on the operative side. All patients underwent the standard Oxford UKA surgical procedure (minimally invasive Oxford UKA, using the Oxford Microplasty instrumentation). All patients received an analgesic intra-articular cocktail mixture injection containing ropivacaine, parecoxib sodium, oxycodone, epinephrine, and tranexamic acid. Patients received an intravenous infusion of drugs to control pain for 3 days after surgery. All patients routinely received a drainage tube; removed on the first day after surgery. All patients received anticoagulant therapy from 1 day after surgery until 2 weeks after surgery.

### Outcome measures

We used the Hospital for Special Surgery (HSS) Knee score and Western Ontario and McMaster (WOMAC) Universities Osteoarthritis Index to evaluate the general condition of the patients’ knees. Preoperative and postoperative range of motion (ROM), HSS, and WOMAC score were used to assess patient function at final follow-up. Postoperative radiographs in three groups, collected at the latest follow-up, were reviewed by the authors to identify any signs of radiolucency, loosening, and progression of arthritis in the lateral and patellofemoral compartments.

Depending on the age of the patients, we divided all patients into three groups (ages 60–69; ages 70–79; age over 80). Patient records were reviewed and the following data collected: American Society of Anesthesiologists (ASA) score, body mass index (BMI), type of anesthesia, length of stay, hemoglobin values (before surgery and three days after surgery), change of hematocrit (before surgery, first and third day after surgery), tourniquet time, and previous basic disease (e.g., hypertension, diabetes, coronary heart disease). We used the Mercurialis method to calculate the volume of blood loss [8]. We used the visual analogue scale (VAS) score to evaluate pain degree eight hours (h), 16 h, and 24 h after surgery. We also recorded preoperative and postoperative ROM, HSS, and WOMAC scores as well as perioperative and short-term complications.

### Statistical analysis

The statistical software used for all analyses was SPSS 22.0 (SPSS Inc., Chicago, IL, USA). Continuous variables are reported as means ± standard deviation (with range). Discrete variables are reported as number (percent). Chi-squared test or Fisher’s exact probability method was used to compare binary variables (demographic data and complication rates). We used analysis of variance (ANOVA) for comparison of clinical scores (HSS score, WOMAC score) between the three groups (significance set at *P* < 0.05).

The minimum clinically important differences (MCID) of the WOMAC score was 11 for pain, 9 for function, 8 for stiffness, and 10 for the total WOMAC score [9]. The MCID of HSS score was 6 according to previous study [10]. Our sample size was sufficient to detect potentially relevant differences regarding this clinical parameter.

One-way ANOVA was used for the comparison of the means between the three groups. Homogeneity of variance is needed before conducting ANOVA. First, we calculated a global/overall model. Only in case of significance, Bonferroni adjusted pairwise comparisons are needed to detect which groups differ significantly. The binary variables between the multiple groups is expressed in percentage (P) and was compared using Fisher’s exact probability method or chi-square test.

## Results

One hundred ninety five patients enrolled in our study. Among the patients, 60 (60 knees) were 60–69 years old (Group 1); 70 patients (79 knees), 70–79 years old (Group 2); and 65 patients (70 knees), older than 80 years (Group 3). The mean follow-up was 21.34 ± 12.04, 22.08 ± 11.38, and 21.76 ± 10.20 months in groups 1, 2, and 3, respectively. Table 1 describes the general conditions of all the patients.

**Table 1:** Patient Demographic Characteristics

Variable	Group 1	Group 2	Group 3
Knees	60	79	70
Age (y)	64.93 ± 3.28	75.17 ± 4.12	82.41 ± 2.40
Sex (male: female)	26:34	30:49	34:36
Follow-up (m)	21.34 ± 12.04	22.08 ± 11.38	21.76 ± 10.20

The patients’ characteristics and perioperative variables between different groups are shown in Table 2. In Group 3, the number of patients with hypertension was statistically higher than that of groups 1 and 2 (*p* < 0.05). There were no significant differences in ASA scores, BMI, and other basic diseases between the three groups. There were no significant differences in preoperative ROM.

**Table 2:** Comparison of Patient Characteristics, and Preoperative Variables Between different groups

Variable	Group 1	Group 2	Group 3	F value/χ2	η2	*P* Value
ASA score	1.5 ± 0.5	1.5 ± 0.5	1.5 ± 0.5	0.617	0.012	0.723
1–2	51 (85.0%)	64 (81.0%)	51 (72.9%)			
3–4	9 (15.0%)	15 (19.0%)	19 (27.1%)			
BMI	27.35 ± 3.64	27.19 ± 3.82	26.84 ± 3.61	0.125	0.007	0.932
Normal (< 25)	21 (35.0%)	28 (35.5%)	26 (37.1%)			
Overweight (25–30)	30 (50.0%)	31 (39.2%)	30 (42.9%)			
Obese (> 30)	9 (15.0%)	20 (25.3%)	14 (20.0%)			
Anesthesia						
General	1 (1.7%)	2 (2.5%)	10 (14.3%)			
Spine	59 (98.3%%)	77 (97.5%)	60 (85.7%)			
Hypertension	35 (58.3%)	48 (60.7%)	57 (81.4%)*	8.192	/	0.036
Diabetes	18 (30.0%)	24 (30.4%)	19 (27.1%)	0.663	/	0.792
Coronary heart disease	14 (23.3%)	19 (24.1%)	20 (28.5%)	0.386	/	0.697
Digestive diseases	5 (8.3%)	7 (8.8%)	7 (10.0%)	0.286	/	0.711
Nervous system disease	6 (10.0%)	9 (11.4%)	12 (17.1%)	2.861	/	0.323
Immune system disease	1 (1.7%)	2 (2.5%)	0	2.102	/	0.283
Respiratory diseases	2 (3.3%)	3 (3.8%)	7 (10.0%)	3.287	/	0.113
Peripheral vascular disease	1 (1.7%)	2 (2.5%)	6 (8.5%)	1.983	/	0.204
Coagulation abnormalities	8 (13.3%)	12 (15.2%)	11 (15.7%)	0.582	/	0.841

“*” Represent compared with the Group 1and 2, the difference in Group 3 is statistically different

The postoperative variables and perioperative complications between the different groups are shown in Table 3. There were no significant differences in hospital stay, tourniquet time, changes of hemoglobin, blood loss volume, or postoperative ROM between the three groups. Regarding perioperative complications, the risk of superficial wound infection and wound swelling in elderly patients was slightly higher than that in patients under 80 years old (*p* < 0.05). A total of 13 patients suffered superficial infection after surgery. They received vacuum sealing drainage (VSD) and recovered. There were no significant differences in other perioperative complications.

**Table 3:** Comparison of postoperative variables and Perioperative complications between different groups

Variable	Group 1	Group 2	Group 3	*F value/*χ2	η2	*P* Value
Hospital stay (d)	10.54 ± 3.37	11.23 ± 2.82	12.64 ± 2.68	1.276	0.009	0.387
Tourniquet time (min)	72.50 ± 6.57	75.16 ± 9.53	76.23 ± 11.18	1.785	0.012	0.204
Changes of hemoglobin (g/L)	14.11 ± 7.48	15.29 ± 9.39	13.05 ± 8.69	0.967	0.026	0.458
Blood loss volume (ml)	178.17 ± 74.73	179.80 ± 84.22	175.50 ± 80.39	0.641	0.034	0.783
Preoperative ROM	111.91 ± 11.57	108.83 ± 10.84	103.86 ± 10.68	0.884	0.075	0.539
Postoperative ROM	120.23 ± 7.99	115.38 ± 8.27	112.95 ± 7.01	0.923	0.115	0.413
Perioperative complications						
Myocardial infarction	0	0	0			
Congestive heart failure	1 (1.6%)	0	0	0.598	/	0.567
Cerebrovascular accident	0	0	0			
Lung infection	1 (1.6%)	1 (1.2%)	2 (2.9%)	0.682	/	0.542
Pulmonary embolism	0	0	0			
Urinary system infection	0	0	0			
Abnormal renal and liver function	2 (3.3%)	4 (5.1%)	0	3.117	/	0.195
Deep vein thrombosis	3 (5.0%)	5 (6.3%)	5 (7.1%)	0.265	/	0.838
Calf muscular vein thrombosis	9 (15.0%)	14 (17.7%)	13 (18.5%)	0.386	/	0.643
Hypoproteinemia	8 (13.3%)	9 (11.4%)	11 (15.7%)	1.487	/	0.455
Superficial infection	1 (1.6%)	1 (1.2%)	11 (15.7%)*	11.248	/	0.015
Deep infection	0	0	0			
Swelling of the wound	4 (6.6%)	5 (6.3%)	12 (17.1%)*	10.749	/	0.023

“*” Represent compared with the Group 1and 2, the difference in Group 3 is statistically different

There were also no statistical differences in preoperative and postoperative HSS or WOMAC scores (Table 4). Although there was no statistical difference between the three groups, the knee scores of older patients were still lower than those of the relatively younger patients. Compared to before surgery, significant improvements were found in HSS and WOMAC scores among the three groups. Compared to groups 1 and 2, Group 3 (*p* < 0.05) showed a lower mean function score in HSS and WOMAC scores, but this was improved compared to the preoperative condition. The MCID of the WOMAC score was 10 and the MCID of HSS score was 6 [9, 10]. These differences may be of little importance. There were no significant differences in postoperative VAS scores between the three groups.

**Table 4:** Comparison of preoperative and Postoperative Knee score between different groups

	Group 1	Group 2	Group 3	F Value	η2	*P* Value
VAS score (8 h after surgery)	1.08 ± 0.83	1.29 ± 0.56	1.25 ± 0.57	0.912	0.019	0.608
VAS score (16 h after surgery)	1.65 ± 0.76	1.83 ± 0.79	1.80 ± 0.72	0.823	0.009	0.590
VAS score (24 h after surgery)	1.43 ± 0.48	1.73 ± 0.42	1.54 ± 0.56	1.023	0.021	0.667
Preoperative HSS	61.74 ± 6.96	58.13 ± 7.59	55.68 ± 7.53	1.468	0.092	0.147
Function score	13.96 ± 3.08	12.59 ± 2.36	11.09 ± 2.60	1.997	0.091	0.160
Pain score	13.20 ± 6.27	13.00 ± 5.28	12.27 ± 4.30	0.921	0.059	0.513
Preoperative WOMAC	44.30 ± 11.26	46.85 ± 14.90	48.91 ± 13.10	1.342	0.134	0.208
Function score	32.28 ± 10.27	33.28 ± 12.27	36.68 ± 11.58	2.864	0.099	0.140
Pain score	11.60 ± 3.86	10.50 ± 4.06	10.88 ± 4.63	0.864	0.091	0.684
Stiffness	3.01 ± 1.98	2.98.50 ± 3.06	3.21 ± 2.11	0.819	0.089	0.798
Postoperative HSS	86.61 ± 6.38	85.23 ± 6.98	83.09 ± 6.04	2.128	0.206	0.129
Function score	19.34 ± 2.56	18.34 ± 2.59	16.64 ± 1.56*	15.736	0.125	< 0.001
Pain score	5.96 ± 2.08	5.39 ± 3.14	5.76 ± 3.08	0.866	0.085	0.785
Postoperative WOMAC	24.16 ± 10.53	25.56 ± 10.53	28.00 ± 9.50	3.437	0.233	0.086
Function score	14.28 ± 5.53	13.89 ± 4.69	18.23 ± 5.81*	10.289	0.134	< 0.001
Pain score	5.29 ± 3.19	5.46 ± 4.42	5.76 ± 3.08	0.854	0.104	0.781
Stiffness	2.26 ± 1.36	1.98 ± 1.43	2.24 ± 1.14	1.876	0.201	0.620
Change of HSS	24.87 ± 6.67	27.10 ± 6.83	27.40 ± 6.02	2.145	0.001	0.108
Change of HSS function score	5.68 ± 3.21	6.21 ± 3.02	5.85 ± 4.15	0.987	0.018	0.698
Change of WOMAC	20.93 ± 4.82	21.90 ± 5.39	20.91 ± 4.68	0.853	0.021	0.728
Change of WOMAC function score	19.18 ± 8.01	20.08 ± 6.99	18.00 ± 7.10	1.256	0.013	0.657

“*” Represent compared with the Group 1and 2, the difference in Group 3 is statistically different

Table 5 describes the surgery-related complications after Oxford UKA. One patient in Group 1 developed loosening of the tibial component 2 years after surgery, but did not have any complaint of discomfort. We are continuing clinical follow-up of this patient. Group 1 had 5 patients found to have radiolucent lines at final follow-up; Group 2, 5 patients; and Group 3, 9 patients. One patient in Group 1 suffered bearing dislocation four months after surgery and subsequently received a TKA. One patient in Group 3 accidentally fell two months after surgery and was diagnosed with a periprosthetic fracture. This patient later underwent open reduction of the tibial fracture with internal fixation (LCP Medial Proximal Tibial Plate).

**Table 5:** The surgery-related complications after Oxford UKA

Complication	Group 1	Group 2	Group 3
Implant loosening	1	0	0
Radiolucency	5	5	9
Dislocation	1	0	0
Periprosthetic fracture	0	0	1
Periprosthetic joint infection	0	0	0
Progression of arthritis	0	0	0
Persistent unexplained pain	4	2	3

## Discussion

There were no major systemic complications following 209 consecutive Oxford UKAs (195 patients). This study revealed no significant differences in the perioperative complication rates in patients between the different groups. All patients obtained satisfactory clinical outcomes, but compared to patients over 80 years old, patients between the ages of 60–79 had a higher function score in both HSS and WOMAC scores.

### Clinical outcome of UKA

Iacono et al. [11] evaluated results obtained in patients older than 75 years treated with UKA. All clinical scores improved significantly at follow-up, and the outcome was considered good or excellent in 92.6% of the patients, but the prosthesis used was different from ours.

Concerning the clinical outcomes in very old patients who underwent Oxford UKA, a recent study from the Oxford center analyzed 1000 Oxford UKAs and found that at 10-year follow-up, patients younger than 60 at the time of the operation had significantly better American Knee Society Score Function (AKSS-F) score, Oxford Knee Score (OKS), and Tegner Activity Score than patients older than 60, but no difference in functional outcomes was seen between the groups [12]. A meta-analysis reported that the functional outcome of UKA in the elderly is good, with low rates of perioperative morbidity and mortality [13]. Inale et al. [14] reviewed the short-term results of mobile-bearing medial UKA in elderly patients and compared the results with younger patients. The differences between the knee scores from the elderly patients and from the younger patients were not statistically significant. Revision rate and survival of the implant were not different among the groups.

In our study, there was a clear improvement in HSS and WOMAC scores in both groups after surgery. WOMAC scores evaluate efficacy through three aspects: function, pain, and stiffness. HSS scores evaluate efficacy through two aspects: function and pain. There was no statistical difference between the three groups in the total HSS score and WOMAC score. However, Group 3 had lower scores in the functional dimension in HSS and WOMAC scores, and there was no statistical difference in the pain aspects in HSS and WOMAC scores. According to previous study [9, 10], the MCID of the WOMAC score and HSS was 10 and 6.This is considered small and still within the MCID of the function outcome measurement. Thus, even though the difference is statistically significant, it might not be clinically important. The lack of exercise and the decline of activity by patients older than 80 might also have led to this finding.

In Asian populations, body size, BMI, lifestyle, and knee morphology of Asian populations differ from those in Western countries. A proportion of patients, whose knees flex more than 120 degrees, are required to perform daily activities that include squatting and sitting on the floor, which may lead to different clinical outcomes from Western populations. Lim et al. reported that Oxford UKA can yield satisfactory clinical and functional results and has a 10-year survival rate of 94% in Korean patients [7]. Yoshida et al. report similarly good medium-term results with a 10-year survival rate of 95% in Japanese patients [15]. In our study, the clinical outcome of Chinese patients is similar to Western patients’ clinical outcome, and the ROM also changes significantly in both groups before and after surgery.

### Surgery-related complications

**Fig. 1:**
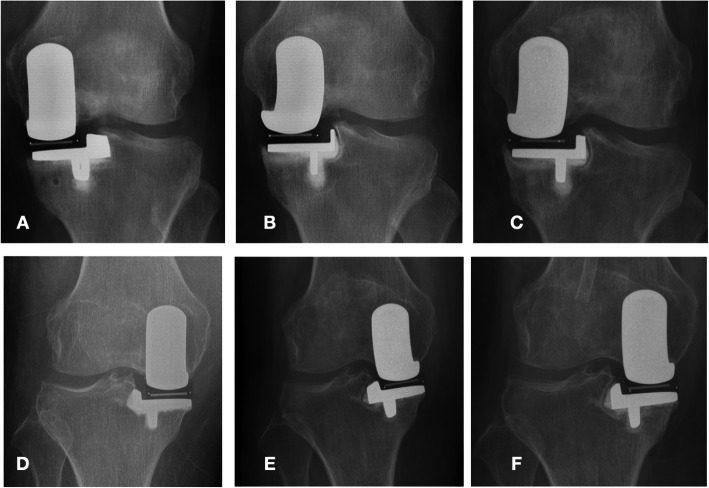
shows 2 patients developed radiolucent line after surgery (6 months after surgery, 1 year after surgery), both the 2 patients did not have pain or other symptoms. **a**, **b** and **c** are the X-rays of the patient 3 months after surgery, 1 year after surgery and 2 years after surgery. **d**, **e** and **f** are the X-rays of another patient 2 weeks after surgery, 6 months after surgery and 1 year after surgery

One systematic review assessed over 8000 Oxford UKA patients and found the 10-year survival to be 93%, 15-year survival to be 89%, and a medical complication incidence of 0.8%. Very good outcomes were achieved by both designing and non-designing surgeons [16]. The literature shows that the main reasons that led to failures of Oxford UKA were bearing dislocation, aseptic loosening, lateral compartment arthritis progression, and persistent unexplained pain [16–20]. Of the 209 Oxford UKAs in our study, 19 (9.1%) patients were found to have radiolucent lines (RLL) under the tibial component on radiographs at final follow-up. This is different from the results reported by other studies. Previous literature shows that the incidence of RLL ranged from 62 to 96%, which was not clinically related to inferior functional outcomes [21–24]. The etiology of radiolucency remains unknown. The incidence of RLL in the current study was lower than in the previous literature. Several reasons may lead to this phenomenon, such as the small sample size, short follow-up time, and lack of standard X-rays (we could usually not get standard X-rays for outpatient follow-ups). Goodfellow et al. describe pathological RLL being > 2 mm thickness, poorly defined, and often related to aseptic loosening. On the contrary, physiological RLL are 1–2 mm thick, and well-defined [25]. The presence of RLL in our patients was not related to their symptoms or indicative or predictive of loosening and, according to the X-rays, we confirmed them to be physiological RLL (Fig. 1). We still need to assess the clinical outcomes through mid- and long-term follow-ups.

**Fig. 2:**
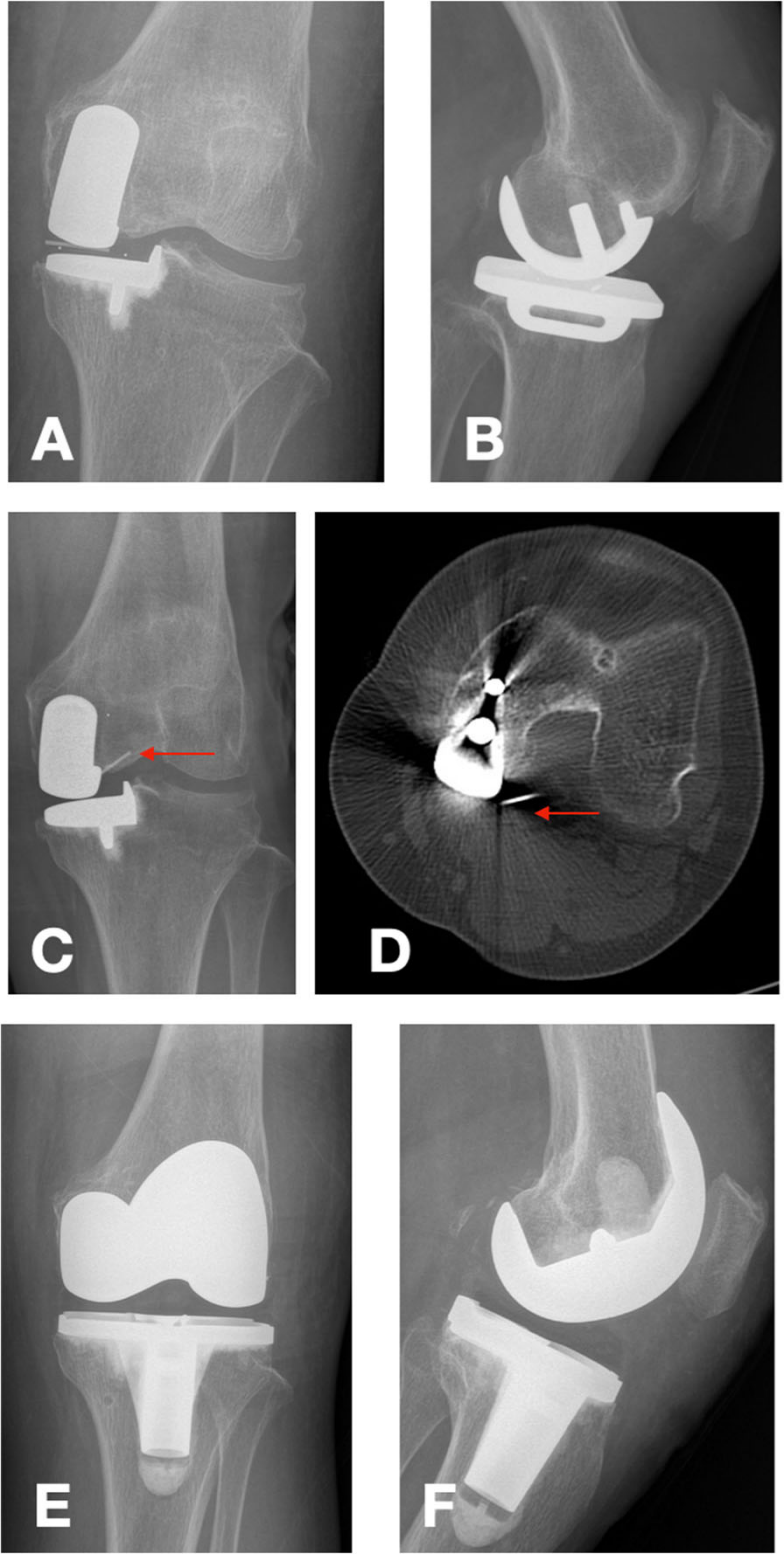
shows a 64-year-old woman suffered moderate pain 4 months after surgery, and the patients received X-ray and CT scan for examination. From the data of imaging we found bearing dislocation. Later the patient performed TKA and got satisfactory clinical outcome after TKA. **a** and **b** are the X-rays of the patient 2 weeks after surgery. **c** is the X-ray of the patient 4 months after surgery. **d** is the CT scan of the patient 4 months after surgery. **e** and **f** are the X-ray of the patient 1 year after TKA surgery

One patient suffered a bearing dislocation four months after surgery. Figure 2 shows the imaging before and after surgery. The possible reason for this may be that the abnormal morphology of the patient’s femur lead to a deviation in the intramedullary rod positioning and the femoral prosthesis was placed medially. The poor prosthesis position caused rotation of the bearing during knee flexion, resulting in dislocation of the bearing. Bearing dislocation is a major complication of Oxford UKA, as previous literature has reported, and the rate of bearing dislocation is higher in Asian populations than in Western populations [5, 26, 27]. It can occur in the presence of an unbalanced flexion-extension gap, impingement of the bearing with adjacent bone or the tibial/femoral component, instability of the medial compartment due to MCL injury, or secondary to femoral/tibial component loosening [5].

**Fig. 3:**
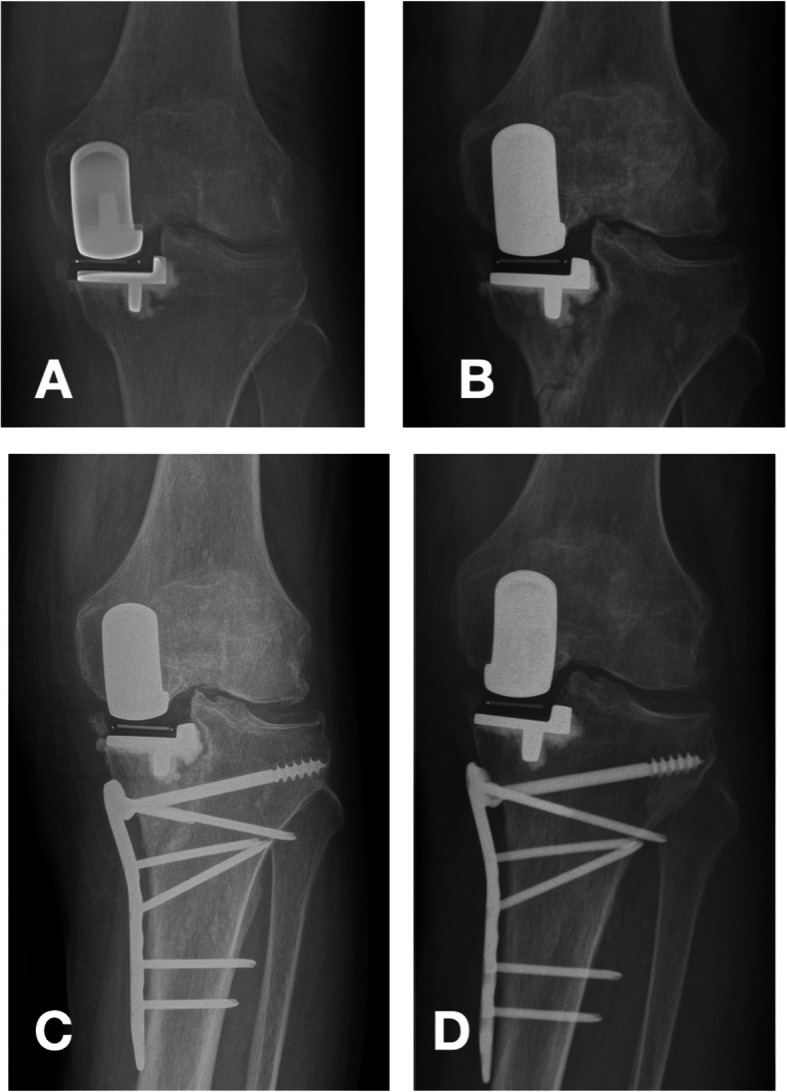
shows an 81-year-old woman fell down 2 months after surgery and later performed open reduction of tibial fracture with internal fixation. **a** is the X-rays of the patient 2 weeks after surgery. **b** is the X-rays of the patient 2 months after surgery. **c** is the X-ray of the patient 3 days after plate fixation. **d** is the X-ray of the patient 8 months after plate fixation

One patient (81 years old) developed a periprosthetic fracture 2 months after surgery due to a fall, and we performed an open reduction of the tibial fracture with internal fixation. Figure 3 shows the X-ray before and after surgery. The literature shows that the rates of fracture in knee arthroplasties are reported to be from 0.2–2.5% in clinical studies and 0.02–0.17% in worldwide arthroplasty registers [28]. Risk factors associated with unicompartmental component periprosthetic fracture include malalignment with increased local stresses due to malpositioning, progressive osteoarthritis, and cruciate ligament deficiency. Patients with a BMI greater than 30 are also at greater risk [29].

### Perioperative complications

The major perioperative complications in our study were calf muscular vein thrombosis (CMVT) and superficial infection. There were no deaths during the perioperative period, pulmonary embolisms, or symptomatic deep vein thrombosis (DVT).

Chan et al. compared one-stage and two-stage bilateral unicompartmental knee replacements during the first 30 days postoperatively and found that the rates of proximal DVT, pulmonary embolus, and death secondary to pulmonary embolus to be 0.9, 1.9, and 0.3%, respectively [30]. If the patient was diagnosed with a DVT or CMVT, they should receive low molecular weight heparin (nadroparin 0.4 mL, twice per day) for 2 weeks. Two weeks after surgery, patients were treated with Rivaroxaban for anticoagulant therapy. The deep vein ultrasound of the lower extremity should then be checked and the drug stopped if the thrombus disappears or dissolves. Other patients received low molecular weight heparin (nadroparin 0.4 mL once per day) after surgery. In total, 13 patients developed superficial infection after surgery (11 patients older than 80). Patient-related risk factors included previous revision arthroplasty, previous infection associated with a prosthetic joint at the same site, tobacco use, obesity, rheumatoid arthritis, a neoplasm, immunosuppression, and diabetes mellitus [31]. Postoperative risk factors included incision healing complications (e.g., superficial infection, hematoma, delayed healing, incision necrosis, and dehiscence), atrial fibrillation, myocardial infarction, urinary tract infection, and prolonged hospital stay [31, 32].

We conclude that Oxford UKA is a safe procedure with a low rate of perioperative complications, similar to previous studies [33]. Previous studies also showed that increased patient age and history of cardiovascular disease were identified as risk factors for perioperative death in TKA [34]. However, in our study, patients older than 80 who underwent Oxford UKA also showed good clinical outcomes with a low rate of perioperative and other complications.

### Limitations

There are several limitations in our study. The study sample was relatively small, and the follow-up relatively short. Further research, large samples, and long-term follow-up are required to evaluate function. The mean follow-up time of the study group in the present study was 21.76 months, which is comparatively long-term if the entry age of 80 years is considered. Moreover, we did not consider the potential influence of sex, and there was no control group in this study.

## Conclusion

Oxford UKA is an effective and safe treatment for osteoarthritis, even in elderly adult patients in China. The knee joint pain symptoms of elderly patients are relieved, and the function improved but was still poor compared with younger patients. The rate of perioperative and other complications in the elderly patients was almost the same as in younger patients. Our study shows the safe use of Oxford UKA in octogenarians in China.

## Data Availability

The datasets analyzed during the current study are available from the corresponding author on reasonable request.

## Abbreviations

ACL: Anterior cruciate ligament
AKSS-F: American Knee Society Score Function score
AMOA: Anteromedial osteoarthritis
ANOVA: Analysis of variance
AP: Anteroposterior
ASA: American Society of Anesthesiologists
BMI: Body mass index
CDFI: Color Doppler flow imaging
CMVT: Calf muscular vein thrombosis
DVT: Deep vein thrombosis
HSS: Hospital for Special Surgery knee score
MCID: Minimum clinically important difference
MCL: Medial collateral ligament
MRI: Magnetic resonance imaging
OKS: Oxford Knee Score
RLL: Radiolucent lines
ROM: Range of motion
TKA: Total knee arthroplasty
UKA: Unicompartmental knee arthroplasty
VAS: Visual analogue scale score
VSD: Vacuum sealing drainage
WOMAC: Western Ontario and McMaster (WOMAC) Universities Osteoarthritis Index
